# COVID-19 dynamics in an Ohio prison

**DOI:** 10.3389/fpubh.2023.1087698

**Published:** 2023-03-30

**Authors:** Wasiur R. KhudaBukhsh, Sat Kartar Khalsa, Eben Kenah, Gregorz A. Rempała, Joseph H. Tien

**Affiliations:** ^1^School of Mathematical Sciences, The University of Nottingham, Nottingham, United Kingdom; ^2^Wexner Medical Center, The Ohio State University, Columbus, OH, United States; ^3^Division of Biostatistics, The Ohio State University, Columbus, OH, United States; ^4^Division of Biostatistics, Department of Mathematics, The Ohio State University, Columbus, OH, United States; ^5^Division of Epidemiology, Department of Mathematics, The Ohio State University, Columbus, OH, United States

**Keywords:** SARS-CoV-2, correctional facilities, mathematical modeling, mass testing, reproduction number

## Abstract

Incarcerated individuals are a highly vulnerable population for infection with severe acute respiratory syndrome coronavirus 2 (SARS-CoV-2). Understanding the transmission of respiratory infections within prisons and between prisons and surrounding communities is a crucial component of pandemic preparedness and response. Here, we use mathematical and statistical models to analyze publicly available data on the spread of SARS-CoV-2 reported by the Ohio Department of Rehabilitation and Corrections (ODRC). Results from mass testing conducted on April 16, 2020 were analyzed together with time of first reported SARS-CoV-2 infection among Marion Correctional Institution (MCI) inmates. Extremely rapid, widespread infection of MCI inmates was reported, with nearly 80% of inmates infected within 3 weeks of the first reported inmate case. The dynamical survival analysis (DSA) framework that we use allows the derivation of explicit likelihoods based on mathematical models of transmission. We find that these data are consistent with three non-exclusive possibilities: (i) a basic reproduction number >14 with a single initially infected inmate, (ii) an initial superspreading event resulting in several hundred initially infected inmates with a reproduction number of approximately three, or (iii) earlier undetected circulation of virus among inmates prior to April. All three scenarios attest to the vulnerabilities of prisoners to COVID-19, and the inability to distinguish among these possibilities highlights the need for improved infection surveillance and reporting in prisons.

## 1. Introduction

The COVID-19 pandemic has demonstrated the tremendous vulnerability of incarcerated individuals to respiratory infections. More than 600,000 COVID-19 cases and close to 3,000 deaths were reported among incarcerated individuals in the United States as of October 2022 (1), and case rates for incarcerated individuals are more than five times higher than for the general population (2). Factors contributing to SARS-CoV-2 transmission in prisons include shared housing, crowding, hygiene challenges, and inability to social distance (3). Outbreak sizes within facilities can be high: infections in more than 80% of prisoners at the Marion Correctional Institution (MCI) in Ohio have been identified (4–7), and similarly high levels of infection have been observed at correctional facilities in other jurisdictions (8, 9). The vulnerability of prisoners and prison staff to COVID-19, the epidemiological connections between prisons and between prisons and surrounding communities, and the potential for prisons to become amplifiers of transmission have been noted by many authors (10–21).

Studies of COVID-19 outbreaks in correctional facilities can help us understand transmission in prisons and jails and identify practices to prevent and control future outbreaks. Research during the COVID-19 pandemic addressed vaccine efficacy and uptake studies among inmates and staff (22, 23), examination of policies by state corrections departments (24), studies of outbreaks started by transfer of infected inmates between prisons (20), quantitative analyses of relationships between correctional facility cases and cases in surrounding communities (18, 19), and analysis of interventions such as decarceration, single-celling, and testing of asymptomatic individuals (25). Analyses of outbreaks in specific facilities include time series analysis (9), assessment of outbreak response in a California state prison (16), and network analyses based upon inmate housing and staff assignments (26). Modeling studies include stochastic simulations of transmission among inmates and staff (27) and fitting compartmental models to case time series data (28). In particular, Puglisi et al. (28) use model fits to estimate the basic reproduction number (*R*_0_) for the ancestral strain of SARS-CoV-2 in a large urban jail. Several of these studies point to the need for improved data collection and reporting (21).

Here, we study the COVID-19 outbreak in MCI in the spring of 2020 using publicly available time series data from the Ohio Department of Rehabilitation and Corrections (ODRC). In particular, our main contribution is a rigorous and statistically principled analysis of the results of mass testing conducted at MCI in April 2020. The analysis is based on a compartmental mathematical model of transmission that is fit to data using a statistical approach called the dynamical survival analysis (DSA) (29, 30), which allows the calculation of explicit likelihoods to summarize uncertainty. Our results highlight the explosive potential for transmission of respiratory infections in prisons as well as the critical need for improved monitoring and reporting of infection in correctional facilities.

## 2. Data and methods

### 2.1. Case data

Mass RT-PCR testing of all inmates and partial testing of staff at MCI was conducted on April 16, 2020. The total number of inmates and the number of inmates and staff testing positive for SARS-CoV-2 over time were obtained from public ODRC reports (7). Results from early SARS-CoV-2 tests were available with a slight time-lag, so we accumulate the cases reported at MCI over April 16–23, 2020 as a single mass testing data point assigned to April 16, which was the date of mass testing. The mass testing event received significant media coverage and was reported widely in numerous news articles (4–6).

### 2.2. Mathematical model

We use a compartmental susceptible-exposed-infectious-recovered (SEIR) model of SARS-CoV-2 dynamics in MCI. Such compartmental models have been used extensively in the literature because they tend to provide a good approximation to the process of disease spread (31). Assuming a well-mixed population, under the standard SEIR model, the proportions of individuals in the susceptible (*S*_*t*_), exposed (*E*_*t*_), infectious (*I*_*t*_), and recovered (*R*_*t*_) compartments as a function of time *t* satisfy the following system of differential equations:


(1) $$\begin{array}{l} \begin{array}{l} {Ṡ}_{t}=-\beta S_{t}I_{t},\\ {Ė}_{t}=\beta S_{t}I_{t}-\alpha E_{t},\\ {İ}_{t}=\alpha E_{t}-\gamma I_{t},\\ {Ṙ}_{t}=\gamma I_{t}, \end{array} \end{array}$$

where the positive parameters β, α, and γ denote the infection rate, incubation rate, and recovery rate, respectively.

### 2.3. Statistical analysis

We derive a likelihood function for observing *n* positives out of *N* incarcerated individuals on day *u* as follows: Using the DSA approach of (29, 30, 32, 33), we interpret *S*_*t*_ as an improper survival function. The mathematical justification for such an interpretation is provided by the Sellke construction by which the function *S*_*t*_ can be identified as the limiting probability of an initially susceptible individual not getting infected by time *t*. Note that the function *S*_*t*_ satisfying (1) is indeed a decreasing function and, when properly scaled, we set *S*_0_ = 1. However, unlike proper survival functions that vanish at infinity (i.e., decrease to zero in the limit), the function *S*_*t*_ → *S*_∞_ > 0 as *t* → ∞ so it is an improper survival function. However, we make it a proper survival function by conditioning on ever being infected. Given observation up to time *T* > 0, the time *T*_*E*_ that an initially susceptible individual becomes infected and enters the *E* compartment follows the conditional probability density function


(2) $$\begin{array}{l} f_{T}\left(t\right)=-\frac{{Ṡ}_{t}}{\tau_{T}}, \end{array}$$

where τ_*T*_ = 1 − *S*_*T*_. The time *T*_*I*_ to becoming infectious has the conditional density


(3) $$\begin{array}{l} g_{T}\left(t\right)=\frac{\alpha E_{t}}{\tau_{t}}, \end{array}$$

and the recovery time *T*_*R*_ has the conditional density


(4) $$\begin{array}{l} h_{T}\left(t\right)=\frac{\gamma \left(I_{t}-\rho e^{-\gamma t}\right)}{\tau_{T}}. \end{array}$$

Note that the random variables *T*_*E*_, *T*_*I*_ − *T*_*E*_, and *T*_*R*_ − *T*_*I*_ are mutually independent and that *T*_*I*_ − *T*_*E*_ and *T*_*R*_ − *T*_*I*_ have exponential distributions with rates α and γ, respectively (29). The parameter ρ is the initial proportion of infectious individuals.

Mass testing yields a number of individuals who test positive and a total number of tests administered on the day of mass testing. To use these data, let *T*_*N*_ denote the time when virus first becomes undetectable in an individual. We then describe the epidemic process by the pair of random variables (*T*_*E*_, *T*_*N*_). Let ε = *T*_*N*_ − *T*_*E*_. Then, the probability of an individual testing positive on the day of the mass testing (at time *u*) is given by


(5) $$\begin{array}{l} p_{u}=\mathrm{Pr}\left(T_{E}<u<T_{N}\right)=\mathrm{Pr}\left(0<u-T_{E}<\varepsilon \right). \end{array}$$

We fix ε = 21 days, corresponding to detectable virus for 3 weeks following an individual becoming infectious (34, 35). We set 1/α = 5.1/log(2) days [corresponding to a median incubation period of 5.1 days (36)] and assume a mean infectious period 1/γ of 5.6 days (37).

If *n* out of *N* individuals test positive on the day of the mass testing *u*, the log-likelihood function is given by


(6) ℓ(β|n,N)=log((Nn)pun(1-pu)N-n),

with the probability of testing positive *p*_*u*_ as described in Equation (5). Note that the above likelihood function is a consequence of the functional law of the large numbers for Poisson processes and the Sellke construction.

The crux of the DSA method is that it allows one to interpret functions that describe the large-population limiting proportions of individuals in different compartments as probabilistic quantities, such as survival functions or probability density functions of transfer times from one compartment to another. This change in perspective has a number of statistical advantages. For instance, it makes available the entire toolkit of survival analysis, by virtue of which it can account for censoring, truncation and aggregation of data in a natural way. Variations of the DSA method have been recently applied to analyze not only COVID-19, but also the 2001 foot-and-mouth disease (FMD) outbreak in the United Kingdom (30) and multiple waves of the 2018–2020 Ebola epidemic in the Democratic Republic of Congo (32). It is important to note that the date considered in this article are from the first phase of the pandemic when vaccines were not yet available. Nevertheless, the DSA method is capable to incorporating vaccination regimes. For instance, the method was applied to assess the potential impact of vaccination in Israel in (38). See also Klaus et al. (39) where the method was applied to COVID-19 data in the state of Ohio, USA.

## 3. Results

### 3.1. Reported outbreak time course

According to ODRC reports (7), the first identified COVID-19 case at MCI was an infected staff member on March 29. Following this initial case, precautions such as cohorting and modified movement were enacted in order to restrict mixing and reduce transmission. As stated in the publicly available ODRC report from March 30, 2020:

Based on a staff member reporting a positive COVID test, MCI is operating under modified movement and the population is being separated by unit along with other precautionary measures. Every inmate at MCI is monitored daily and has their temperature taken along with a check for symptoms. Currently, there are no inmates symptomatic for COVID-19.

**Figure 1:**
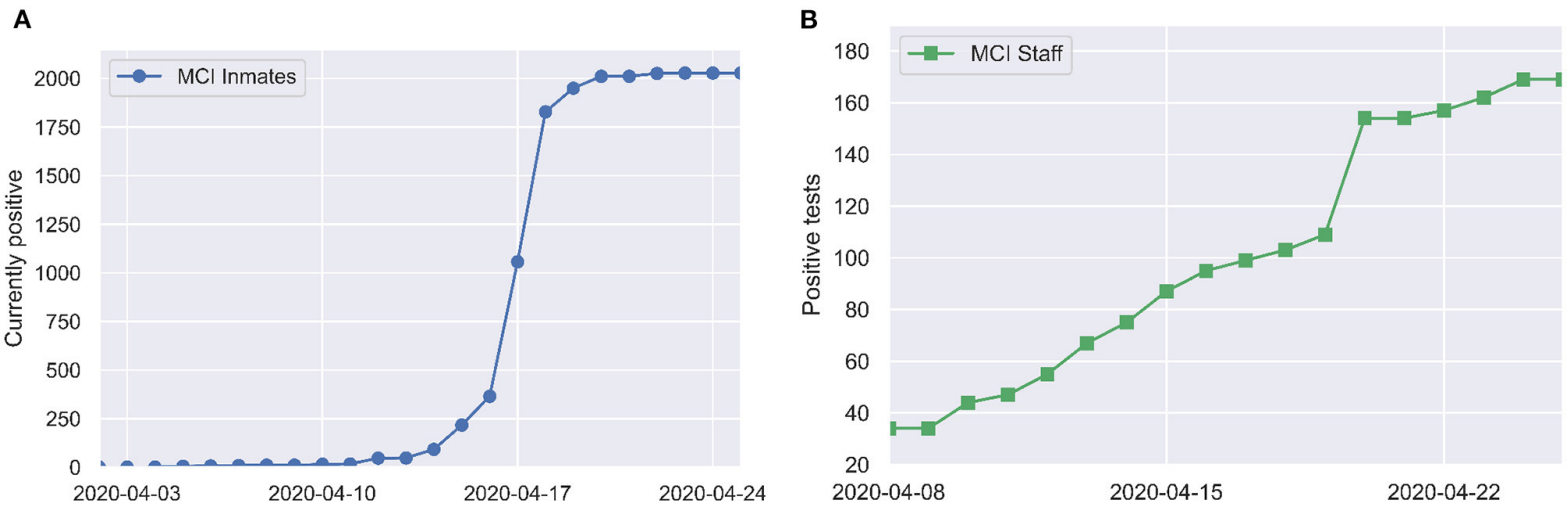
Positive tests over time among **(A)** inmates and **(B)** staff at MCI, as reported by the Ohio Department of Corrections and Rehabilitation (7).

The first COVID-19 case among inmates was identified on April 3. Mass RT-PCR testing of all inmates and partial testing of staff was conducted on April 16. By April 20, SARS-CoV-2 infection had been identified in 79% (1,950/2,453) of inmates and 35% (154/446) of staff. These numbers come directly from data on the ODRC website. We take the May 5, 2020 listing of 2,453 inmates at MCI as the denominator. There is a lag of a few days between when mass testing occurred (April 16) and when jumps in case counts are reported in the ODRC data (April 18–19 for inmates and April 20 for staff), which may reflect delay in data entry. Figure 1 shows a time series of reported COVID-19 cases at MCI.

### 3.2. Basic reproduction number and initial exposure size

The basic reproduction number R_0_ is one of the key parameters in models of infectious diseases (31). It is defined as the expected number of secondary cases generated by an infected individual in a population where all individuals are susceptible to infection. When R_0_ > 1, disease can spread rapidly and cause a large epidemic with positive probability. When R_0_ < 1, the spread of disease dies out stochastically and a large epidemic cannot occur. R_0_ can also be used to calculate the so-called “herd immunity threshold” for interventions like vaccination that effectively reduce the susceptible population.

**Figure 2:**
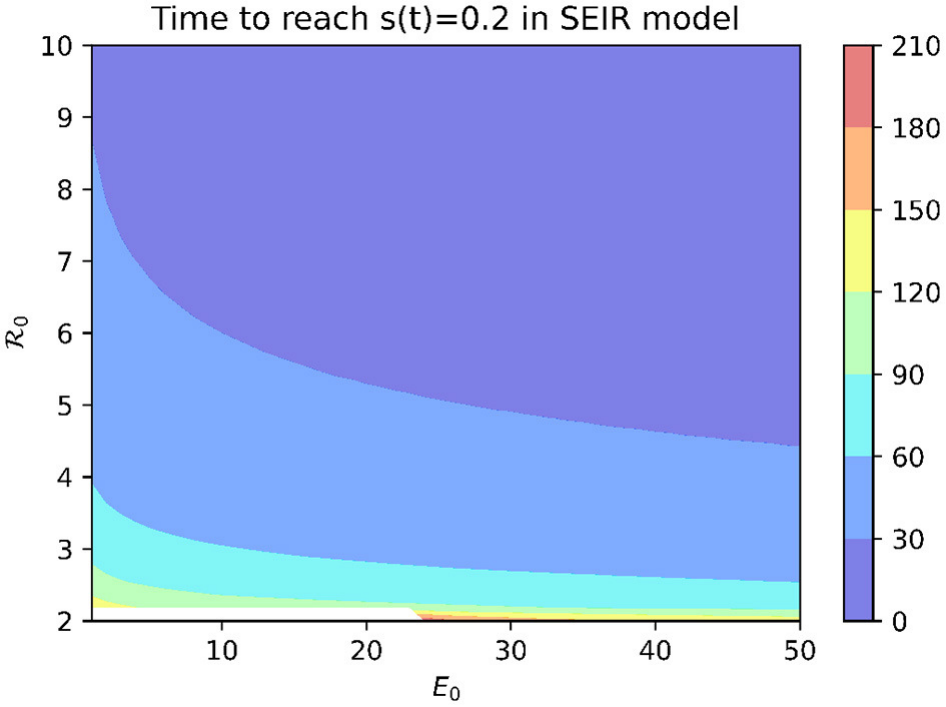
Time to infect 80% of the population in SEIR model with median incubation period of 5.1 days and mean infectious period of 5.6 days.

To examine which values of the basic reproduction number R_0_ are consistent with the rapid spread of COVID-19 observed at MCI, we use the SEIR model (1). In order for mass screening to identify 80% of the population as positive for COVID-19 on April 16, at least 80% of the population must have been infected by that date. Figure 2 shows the time needed to infect 80% of the population in the SEIR model as a function of R_0_ and the initial number of exposed individuals (*E*_0_). While R_0_ values of two or larger are able to eventually infect 80% or more of the population, this can take on the order of months for modest values of R_0_. Reproduction numbers >14 are needed before outbreaks originating from a single exposed individual are able to generate a 3-week cumulative incidence consistent with that reported for MCI.

An alternative explanation is that the outbreak involved more than one initially infected prisoner. Figure 2 shows that, for a fixed R_0_ value, increasing *E*_0_ decreases the time needed to infect 80% of the population. However, an initial condition of *E*_0_ > 563 is needed for an outbreak with R_0_ = 3 to infect 80% of the population within 3 weeks.

### 3.3. Time of initial outbreak circulation

**Figure 3:**
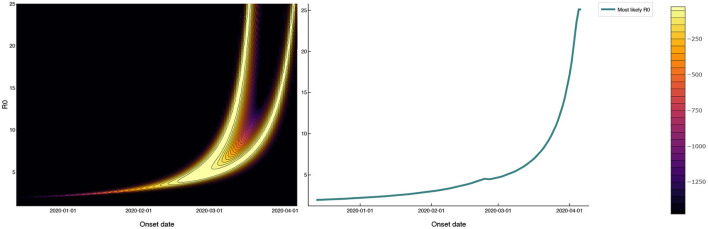
Log-likelihood in the (R_0_, outbreak onset date) plane for mass testing data under a SEIR model with a 3 week test-positive window following the onset of infectiousness. The R_0_ values that maximize the likelihood as a function of outbreak onset date are shown as a solid red line that follows the right-most branch of the ‘wishbone' in the log-likelihood plot. The R_0_ values consistent with the reported data (with onsets in late March or later) are as large as 10. Onset dates prior to March, while inconsistent with the reported data, give more realistic R_0_ values of less than five.

A third possibility is that SARS-CoV-2 was circulating among prisoners prior to April 3. Figure 3 shows the log-likelihood (6) for observing the mass testing results in MCI according to the SEIR model (1) as a function of R_0_ and the outbreak onset date, with *E*_0_ fixed at one. The outbreak onset date and R_0_ are unidentifiable from the mass testing data alone, with the “wishbone” shape running diagonally across Figure 3 corresponding to pairs of outbreak onset and R_0_ that are almost equally likely given the observed data. Outbreak onsets in late March or later correspond to R_0_ > 10, while earlier outbreak onsets correspond to smaller R_0_ values. Note that onset dates prior to March are required to give R_0_ values of less than five.

In general, the larger the value of the parameter R_0_, the more difficult it is to control the epidemic. Our analysis is consistent with this. Both the first and the third possibilities explained above suggest that the R_0_ values consistent with the reported data must be extremely high, calling attention to the explosive potential for COVID-19 transmission in prisons. Both the second and the third possibilities underscore the implausibility of the reported disease introduction date and/or the initial amount of infection, calling attention to the need for more reliable monitoring and reporting of infection in correctional facilities.

## 4. Discussion and conclusion

The official reports from ODRC describe widespread infection of MCI inmates with SARS-CoV-2 within the span of 3 weeks. Our primary contribution is a rigorous analysis of the data using an SEIR compartmental model fit to these data using the DSA approach, which allows us to use all of the tools of likelihood-based inference. This analysis indicates three non-exclusive possible explanations for this rapid spread: (i) values for the basic reproduction number that are far higher than the R_0_ values between two and three that have been estimated for the ancestral strain of SARS-CoV-2 in non-prison settings in the United States (37), (ii) initial exposure of a large number of infected prisoners as in an extreme superspreading event, or (iii) early undetected circulation of SARS-CoV-2 among prisoners prior to April 3. We note that the R_0_ values in (i) are even greater than the already high estimates of the basic reproduction number in a large urban jail (28). All three possibilities speak to the vulnerabilities of prison inmates and staff to COVID-19. Distinguishing between these different scenarios is impossible without improved data collection and reporting. An arguable weakness of our analysis is that it is retrospective in nature. However, we believe studies such as ours will lead to improvements that allow more detailed insight into the transmission of respiratory infections within prisons are critical for protecting the health of prison inmates, staff, and surrounding communities in future pandemics.

Permissive conditions for spread within correctional facilities, challenges for disease surveillance and care in these settings, and the inextricable link between COVID-19 within correctional facilities and disease spread in the surrounding community, have been discussed eloquently by others (10–14). Structural changes such as lower inmate densities (25, 40) and improved ventilation (9, 16) are needed to decrease transmission potential in correctional facilities. Efforts to increase vaccine coverage are also important, particularly among prison staff who may have relatively low vaccine uptake (23). Community case rates are associated with cases in prisons (18), inmate transfers can allow outbreaks to jump from one prison to another (20), and staff can be an epidemiological link between correctional facilities and surrounding communities. Without changes to protect the health of staff and inmates, it is predictable prisons will be vulnerable to extremely rapid spread of future respiratory pathogens.

Improved surveillance and reporting are critical for pandemic preparedness and for preventing or controlling future outbreaks of respiratory diseases in prisons. Testing policies during the COVID-19 pandemic varied widely across state corrections departments (24). Testing protocols changed over time, and state reporting of COVID-19 cases in prisons was often incomplete or absent (41). Swift response is essential for preventing and controlling large outbreaks, and it has been identified as a distinguishing feature for countries with successful COVID-19 pandemic responses (42). This swift response is impossible without pathogen detection and reporting efforts that include correctional facilities. Going forward, we urge health departments and corrections departments to collect accurate data and to make these data available for analysis with appropriate protections for human subjects in this vulnerable population.

## Data availability statement

Publicly available datasets were analyzed in this study. This data can be found at: https://github.com/wasiur/PrisonCOVID19Analysis.

## Author contributions

SK collected and organized the data. WK, EK, GR, and JT developed the analytical approach. WK carried out the analysis. All authors contributed to the conception and writing of the manuscript and approved the submitted version.
